# Effects of fetal famine exposure on the cardiovascular disease risk in the metabolic syndrome individuals

**DOI:** 10.1186/s13098-022-00948-0

**Published:** 2022-11-17

**Authors:** Zhe Shu, Xiong Ding, Qing Yue, XiaoXu Ma, MinHong Liu, YunTao Wu, Peng Yang, Ying Wu, Yun Li, Shouling Wu

**Affiliations:** 1 School of Public Health, North China University of Science and Technology, Tangshan, China; 2 School of Public Health, Wuhan University, Wuhan, China; 3 Department of Cardiology, Kailuan General Hospital, 57 Xinhua East Rd, Tangshan, 063000 China; 4 Department of Neurosurgery, Affiliated Hospital of North China University of Science and Technology, Tangshan, China

**Keywords:** Cardiovascular disease, China famine, Metabolic syndrome, Cohort study, Fetal exposure

## Abstract

**Background:**

Patients with metabolic syndrome (MS) have a higher incidence of cardiovascular disease (CVD), but the possible mechanisms are not fully understood and further exploration of the possible factors influencing the high incidence of CVD in patients with MS is still needed.

**Objectives:**

This study aims to examine the association between fetal famine exposure and the risk of CVD in adulthood with MS.

**Methods:**

Of 13,744 MS patients free of CVD selected from the Kailuan Study in 2006 (referred as the baseline survey) were included in the study. China suffered a severe famine from 1959 to 1962, so the participants born during this period were classified as the uterine famine exposed group. All patients were born between January 1, 1949, and December 31, 1974. Based on the date of birth, all patients were divided into the no-exposed group (born between January 1, 1963, and December 31, 1974), uterine famine exposed group (born between January 1, 1959 and December 31, 1962), and childhood famine exposed group (born between January 1, 1949 and December 31, 1958). After following up to December 31, 2019, the weighted Cox regression analysis model was used to calculate the effect of early life famine exposure in MS individuals on the risk of CVD in adulthood.

**Results:**

During the 12.12 years of follow-up, the incidence of CVD was 5.87%, 10.13%, and 10.90% in the no-exposed group, uterine famine exposed group, and childhood famine exposed group, respectively. Compared with participants in the no-exposed group, the CVD risk and stroke risk increased in participants in the uterine famine exposed group (for CVD, HR: 1.32, 95% CI 1.04–1.67; for stroke, HR:1.37, 95% CI 1.05–1.79), but not in childhood famine exposed group. However, the increased CVD risks were only observed in females or smokers. No increased MI risks were observed for participants in the uterine famine exposed group or childhood famine exposed group.

**Conclusions:**

Our findings suggested that exposure to famine during uterine life might increase the risk of CVD in adulthood in participants with MS.

## Introduction

In recent years, the prevalence of metabolic syndrome (MS) has gradually risen [1]. In Chinese adults, the prevalence of MS has increased from 9.5% in 2002 to 18.7% in 2010–2012 [2], with 450 million patients. According to an estimate by the International Diabetes Federation, the worldwide prevalence of MS in adults is on the rise with an estimated prevalence of 20–25% [3]. As is well known, MS could increase the risk of chronic disease, including cardiovascular disease (CVD). Previous studies showed that MS is associated with a twofold increase in CVD [4], and showed that MS increases the risk of CVD over an average of 11 year [5]. Although previous studies [6–9] have suggested that hypertension, hyperglycemia, smoking and physical inactivity might attribute to the high incidence of CVD in MS patients, the etiology was still unclear, and possible influencing factors still need to be explored, which should be helpful in reducing the disease burden of CVD in patients with MS.

"Developmental origins of health and disease" hypothesis suggested that exposure to malnutrition during early life would affect the health in adulthood [10, 11]. Some studies have confirmed that exposure to famine in the uterine period might increase the risk of MS [12], cerebral hemorrhage [13], diabetes [14], hypertension [15], cerebral infarction [16] and other diseases in adulthood, but few studies have examined whether famine exposure affects CVD risk in MS patients.

The Great Famine in China (1959–1962) was one of the largest famines in human history, resulting in insufficient nutritional supply for a large number of people exposed to the famine environment. Our study is based on the Kailuan Study, a large-scale, individual-based longitudinal cohort study with a decade-long follow-up. The expected results of this study will help to examine the association between exposure to the Great Chinese Famine in early life and the risk of CVD in adults in individuals with MS.

## Methods

### Study participants

The Kailuan Study (accession number: ChiCTR-TNC-11001489) was a functional community individual-based cohort study in Tangshan, China, and the specific study design and procedures can be found in the team's previous studies [17, 18]. The Kailuan Study began in 2006 and included 101,510 adults (81,110 males and 20,400 females) aged 18 years or older, all of them completed standard questionnaires (medical history and lifestyle) between 2006 and 2007, underwent health assessments every two years, including physical examinations (waist circumference (WC), weight, height, and blood pressure measurements) and laboratory tests [lipid assessments, fasting blood glucose (FBG), and serum creatinine (SCr)].

According to the International Diabetes Federation global working definition of MS [19], 14,241 MS patients who born between January 1, 1949, and December 31, 1974, were included. Individuals with missing data (n = 69) on WC, high density cholesterol (HDL), triglycerides, diastolic blood pressure (DBP), systolic blood pressure (SBP), fasting blood glucose (FBG), or those with CVD (n = 428) at the baseline survey in 2006 were excluded [16].

Finally, 13,744 participants were served as the baseline cohort.

### Famine exposure

Since famine in China occurred concentratedly from 1959 to 1962, the period of famine exposed was classified by birth information. Based on the previous Chinese famine research [20], birth year was taken as the basis for classification of famine exposure, all participants were divided into three groups: no-exposed group (born between January 1, 1963 and December 31, 1974), uterine famine exposed group (born between January 1, 1959 and December 31, 1962), childhood famine exposed group (born between January 1, 1949 and December 31, 1958).

### MS definition

MS was defined following the IDF Global Working Definition (IDF criteria) with the following criteria [19]: the presence of central obesity (waist circumference ≥ 90 cm for males or ≥ 80 cm for females), plus any two of following factors: (i) raised triglyceride level: ≥ 1.7 mmol/L (150 mg/dl) or taking triglyceride-lowering medications; (ii) reduced HDL cholesterol: < 1.03 mmol/L for males or < 1.29 mmol/L for females, or on lipid-lowering medications; (iii) hypertension: SBP ≥ 130 mmHg, or DBP ≥ 85 mmHg, or taking antihypertensive medications; and (iv) raised FBG: ≥ 100 mg/dL, or individuals who have been diagnosed with type 2 diabetes.

### Follow-up and CVD

The starting point was defined as the date of completion of the 2006 annual baseline questionnaire and individuals were followed up until December 31, 2019. During follow-up, CVD incidence was assessed annually, and biochemical markers were collected every two years. The outcome event for the study was the first occurrence of a major CVD, which was defined as the composite of stroke and myocardial infarction (MI) [21, 22]. The Hospital Discharge Register and Municipal Social Insurance Institution database were linked to identify the incidence of CVD based on The International Statistical Classification of Diseases and Related Health Problems 10th Revision (ICD-10) (I61 for intracerebral hemorrhagic stroke, I63 for ischemic stroke, and I21 for MI) [21, 23]. These two databases were updated annually based on follow-up and cover information on all participants in the Kailuan Study. An expert panel collected and reviewed annual discharges records from 11 local hospitals to identify patients who were suspected of CVD.

Incident MI was diagnosed based on the World Health Organization’s Multinational Monitoring of Trends and Determinants in Cardiovascular Disease (MONICA) criteria on basis of clinical symptoms and dynamic changes in clinical presentation, cardiac enzymes and electrocardiogram. Incident stroke diagnosed was according to neurological signs, clinical symptoms, and neuroimaging (from CT or MRI) on the basis of the World Health Organization's criteria. Death data were collected from provincial vital statistics offices, as described in previous studies [24].

### Covariates evaluation and measurement

The questionnaire design, anthropometry, and laboratory data testing were the same as the literature published by our research group [18]. The data collected by the research include birth, gender, smoking, drinking, physical activity, education, history of disease and history of medications using. During the survey, professionally trained medical staff completed the physical examination, including the measurement of their height, weight, waist circumference and blood pressure. Height and waist circumference were measured to the nearest 0.1 cm using the disposable tape measure. Weight was determined by using the calibrated portable digital weighing scale with 0.1 kg precision.

To ensure the reliability of the biochemical measurement results, a venous blood sample was obtained from all subjects who fasted for at least 8 h before the measurement, and 5 ml of fasting elbow venous blood was collected between 7 and 9 AM on the day of the physical examination, and the blood sample was centrifuged to obtain the upper serum for FBG values and lipid levels. All operations were performed strictly by the manufacturer's instructions, and blood samples were tested on a Hitachi (7600) automated biochemistry analyzer.

Age was calculated by subtracting the birthday from the beginning date of medical examination. Height and weight were measured to calculate body mass index (BMI) as weight (kg)/height^2^ (m^2^). Drinking was defined as more than 50 g of alcohol intake per day for male or more than 15 g per day for female. Smoking was defined as smoking an average of at least one cigarette per day for more than in the past year. Education was stratified into two levels: junior high school or below, or senior high school or above. Physical activity was defined as exercise ≥ 4 times per week, with the duration of each exercise at least 20 min, and was classified as current, never/former. Hypertension was defined as SBP over 140 mmHg or a DBP over 90 mmHg, or the fact that the patient was taking antihypertensive medications. Diabetes was defined as FBG ≥ 7.0 mmol/L, or the fact that the patient was taking hypoglycemic medications.

### Statistical analysis of data

All data processing and analyses were performed using SAS version 9.4 (SAS Institute, Cary, North Carolina), and R software version 3.6.0 (R Core Team, Vienna, Austria). The database was established through EpiData 3.1, entered by uniformly trained medical staff, and uploaded to the Oracle database of Kailuan General Hospital. All statistical tests were 2-sided, and P < 0.05 was considered statistically significant.

The normal or approximate normal distribution of the continuous variables was represented by $\overline{\chi }\pm s$, the comparison between groups using analysis of variance, the skewed distribution was represented by M (P_25_, P_75_), and the intergroup comparison was used the Kruskal–Wallis test. The percentages described were used categorical variables and compared by Pearson’s chi-square tests.

Person-years of follow-up were calculated from the return date of the baseline questionnaire to the date of CVD diagnosis, death, loss to follow-up (n = 800, 5.82%), or end of follow-up (December 31, 2019) whichever occurred first. The incidence density of CVD in different groups in the MS individuals was calculated by dividing the number of events by the total number of follow-up person-years (1000/person-year), using the Log-rank test for comparison among groups. We used the weighted Cox regression model to analyze the effect of early life famine exposed in the MS individuals on the risk of CVD in adulthood, and the HR (Hazard Ratio) and 95% CI (confidence interval) was calculated [25]. The model adjusted for age, gender, education, smoking, drinking, physical activity, BMI, history of diabetes, history of hypertension, low-density lipoprotein cholesterol, using antihypertensive medications, using antidiabetic medications, and using lipid-lowering medications.

Taking CVD as the dependent variable, and famine exposure as the independent variable, a stratified analysis was carried out by gender, smoking, and drinking. To verify the robustness of the results, a sensitivity analysis was performed after removing the individuals who had CVD incidents within two years or lost-to-review individuals.

## Results

A total of 13,744 participants (10,254 males and 3,490 females) were enrolled in the current study, 1,777 participants had been exposed to the Chinese famine during utero stage, while 8848 participants had been exposed to the famine during childhood stage, respectively. There were significant differences among the three groups in terms of age, gender, BMI, FBG, SBP, DBP, WC, smoking, physical activity, hypertension, diabetes, using antihypertensive medication, and using antihyperglycemic medications (P < 0.001). Compared to the reference group, participants in the uterine famine exposed group were more likely to be female, had diabetes, hypertension, with greater WC, and higher prevalence of using antihypertensive medications and using antihyperglycemic medications (Table 1).

**Table 1:** Basic characteristics of 13,744 MS participants according to the famine exposure

Components	No-exposed (n = 3119)	Uterine exposed (n = 1777)	Childhood exposed (n = 8848)	P-value
Age (year)	40.3 ± 3.2	46.1 ± 1.3	53.1 ± 2.8	< 0.001
Male (%)	80.6	73.4	72.7	< 0.001
BMI (kg/m^2^)	28.2 ± 3.3	27.8 ± 3.4	27.5 ± 3.1	< 0.001
FBG (mmol/L)	6.0 ± 2.0	6.3 ± 2.2	6.3 ± 2.2	< 0.001
SBP (mmHg)	135.3 ± 17.9	138.0 ± 19.1	141.7 ± 19.3	< 0.001
DBP (mmHg)	89.9 ± 12.2	90.1 ± 12.2	90.2 ± 11.5	0.188
WC (cm)	95.0 ± 7.5	95.1 ± 7.9	95.1 ± 7.5	0.964
LDL (mmol/L)	2.4 ± 0.8	2.4 ± 0.8	2.3 ± 0.9	0.104
HDL (mmol/L)	1.4 ± 0.4	1.5 ± 0.4	1.5 ± 0.4	0.001
Education (%)				< 0.001
Low	2320(74.4)	1461(82.2)	7675(86.7)	
High	799(25.6)	316(17.8)	1173(13.3)	
Smoking (n, %)	1269(40.7)	705(39.7)	2901(32.8)	< 0.001
Drinking (n, %)	1511(48.4)	735(41.4)	3082(34.8)	< 0.001
Physical activity (%)	211(6.8)	125(7.0)	1359(15.4)	< 0.001
Hypertension (%)	1890(60.6)	1138(64.0)	6325(71.5)	< 0.001
Diabetes (%)	431(13.8)	359(20.2)	1899(21.5)	< 0.001
Use of antihypertensive medications (%)	365(11.7)	284(16.0)	1879(21.2)	< 0.001
Use of hypoglycemic medications (%)	63(2.0)	57(3.2)	440(5.0)	< 0.001
Use of hypolipidemic medications (%)	44(1.4)	17(1.0)	159(1.8)	0.023

Data were present as n (%), mean ± SD, or median (P_25_, P_75_) according to variable category. Pearson’s chi-square test, ANOVA analysis, or Kruskal–Wallis test was used to compare differences between groups properly

BMI, body mass index; FBG, fasting blood glucose; SBP, systolic blood pressure; DBP, diastolic blood pressure; WC, waist circumference; LDL, low-density lipoprotein; HDL, high-density lipoprotein; TG, triglyceride

**Fig. 1:**
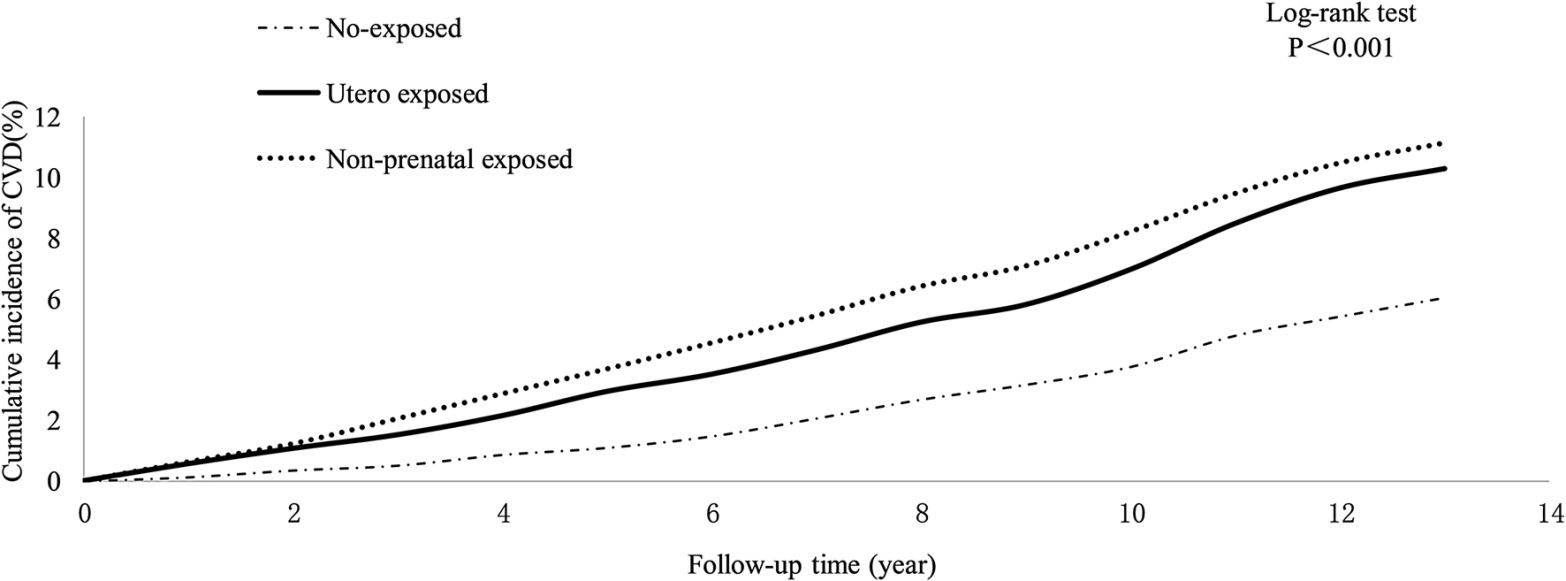
Cumulative incidence curve of CVD in three groups

During a mean follow-up of 12.12 years, the cumulative incidences of CVD in uterine famine exposed group (10.13%) and the childhood famine exposed group (10.90%) were greater than that in no-exposed group (5.87%) (P < 0.05, Fig. 1). The incidence density of CVD was 4.70/1000, 8.32/1000, and 9.09/1000 person-years in the no-exposed group, the uterine famine exposed group, and the childhood famine exposed group, respectively (Table 2). In Table 2 HR for CVD in the uterine famine exposed group in model 1 is 1.41 (1.11–1.78), and in model it is 1.32 (1.04–1.67)—which gives a still significant result, so it could be concluded that the relationship with CVD incidence in the uterine famine exposed group was independent of all covariates included in the last model. In Table 2 HR for stroke in the uterine famine exposed group in model 1 is 1.47 (1.31–1.92), and in model it is 1.37 (1.05–1.79)—which gives a still significant result, so it could be concluded that the relationship with stroke incidence in the uterine famine exposed group was independent of all covariates included in the last model. However, we did not find same association show in MI. In the sensitive analysis, similar results were observed after removing the individuals with CVD occurring within two years or removing the lost-to-review individuals (Table 3).

**Table 2:** Cox proportional hazard model analysis of different famine groups and the incidence of CVD

Components	Case/total	IR (per 1000 person-years)	Model 1 HR(95%CI)	Model 2 HR(95%CI)	Model 3 HR(95%CI)
*CVD*					
No-exposed	183/3119	4.70	1 (Reference)	1 (Reference)	1 (Reference)
Uterine exposed	180/1777	8.32	1.41(1.11–1.78)	1.35(1.06–1.72)	1.32(1.04–1.67)
Childhood exposed	964/8848	9.09	1.10(0.80–1.51)	1.05(0.76–1.45)	1.03(0.75–1.42)
*Stroke*					
No-exposed	145/3119	3.70	1 (Reference)	1 (Reference)	1 (Reference)
Uterine exposed	151/1777	6.94	1.47(1.13–1.92)	1.41(1.08–1.84)	1.37(1.05–1.79)
Childhood exposed	778/8848	7.27	1.10(0.77–1.57)	1.05(0.73–1.51)	1.04(0.72–1.48)
*MI*					
No-exposed	39/3119	0.99	1 (Reference)	1 (Reference)	1 (Reference)
Uterine exposed	33/1777	1.47	1.18(0.69–2.02)	1.16(0.68–1.98)	1.15(0.68–1.97)
Childhood exposed	213/8848	1.94	1.07(0.54–2.12)	1.05(0.53–2.06)	1.05(0.53–2.06)

Model 1: Adjusted for age, and gender

Model 2: Included covariates in model 1 and further adjusted for education (junior high school or below, senior high school or above), smoking (current, never/former), drinking (current, never/former), and physical activity (current, never/former)

Model 3: Included covariates in model 2 and further adjusted for low-density lipoprotein, hypertension, diabetes, use of antihypertensive medications, use of hypoglycemic medications, and use of hypolipidemic medications

HR, hazard ratio; CI, confidence interval; IR, incidence rate; MI, myocardial infarction

**Table 3:** Sensitivity analysis

Components	Case/total	IR (per 1000 person-years)	Model 1 HR(95% CI)	Model 2 HR(95% CI)	Model 3 HR(95% CI)
*Delete events that occurred within 2 years*					
No-exposed	180/3107	4.62	1 (Reference)	1 (Reference)	1 (Reference)
Uterine exposed	173/1762	8.01	1.40(1.10,1.78)	1.35(1.06,1.71)	1.31(1.03,1.67)
Childhood exposed	941/8792	8.88	1.13(0.82,1.56)	1.09(0.79,1.50)	1.06(0.77,1.47)
*Delete lost-to-review individuals*					
No-exposed	176/2981	4.71	1 (Reference)	1 (Reference)	1 (Reference)
Uterine exposed	166/1700	7.95	1.39(1.09,1.78)	1.34(1.05,1.72)	1.31(1.03,1.68)
Childhood exposed	878/8300	8.73	1.14(0.82,1.59)	1.10(0.79,1.53)	1.08(0.78,1.50)

Model 1: Adjusted for age, and gender

Model 2: Included covariates in model 1 and further adjusted for education (junior high school or below, senior high school or above), smoking (current, never/former), drinking (current, never/former), and physical activity (current, never/former)

Model 3: Included covariates in model 2 and further adjusted for low-density lipoprotein, hypertension, diabetes, use of antihypertensive medications, use of hypoglycemic medications, and use of hypolipidemic medications

To evaluate the effect of covariates on the CVD risk, stratified analysis by gender, smoking (yes/no), or drinking (yes/no) were performed. The results showed that the association between uterine famine exposure and increased CVD risk only observed in female (HR: 2.31, 95% CI 1.13–4.73), but not in male. The similar results were observed in smokers (HR:1.53, 95% CI 1.08–2.17). However, no association between famine exposure and CVD risks were observed for patients with childhood famine exposure or drinking. Also, no interaction between famine exposure and gender, smoking or drinking were observed (Table 4).

**Table 4:** Adjusted HR (95% CI) for incidence of CVD in the MS individuals by exposure to famine by gender, smoking, drinking

Components	case/total	IR (per 1000 person-years)	Model 1 HR(95%CI)	Model 2 HR(95%CI)	Model 3 HR(95%CI)	*P* for interaction
*Male*^a^						
No-exposed	168/2514	5.37	1 (Reference)	1 (Reference)	1 (Reference)	0.72
Uterine exposed	148/1304	9.35	1.30(1.01–1.68)	1.25(0.97–1.62)	1.22(0.94–1.58)	
Childhood exposed	807/6436	9.09	1.02(0.73–1.44)	0.98(0.70–1.37)	0.96(0.68–1.35)	
*Female*^a^						
No-exposed	15/605	1.96	1 (Reference)	1 (Reference)	1 (Reference)	
Uterine exposed	32/473	5.52	2.36(1.19–4.67)	2.37(1.20–4.68)	2.31(1.13–4.73)	
Childhood exposed	157/2,412	5.24	1.77(0.69–4.55)	1.84(0.72–4.71)	1.79(0.69–4.65)	
*Smoking*^b^						0.66
No-exposed	91/1269	5.79	1 (Reference)	1 (Reference)	1 (Reference)	
Uterine exposed	92/705	10.84	1.59(1.12–2.25)	1.56(1.10–2.22)	1.53(1.08–2.17)	
Childhood exposed	388/2901	11.37	1.37(0.85–2.21)	1.34(0.83–2.17)	1.33(0.83–2.15)	
*No-Smoking*^b^						
No-exposed	92/1850	3.96	1 (Reference)	1 (Reference)	1 (Reference)	
Uterine exposed	88/1072	6.70	1.26(0.91–1.75)	1.22(0.88–1.70)	1.20(0.86–1.67)	
Childhood exposed	576/5947	8.02	0.94(0.61–1.45)	0.90(0.59–1.39)	0.89(0.58–1.37)	
*Drinking*^c^						0.94
No-exposed	93/1511	4.94	1 (Reference)	1 (Reference)	1 (Reference)	
Uterine exposed	79/735	8.80	1.46(1.02,2.08)	1.38(0.97,1.97)	1.38(0.97,1.97)	
Childhood exposed	363/3082	9.86	1.30(0.81,2.09)	1.23(0.77,1.98)	1.24(0.77,1.99)	
*No-drinking*^c^						
No-exposed	90/1608	4.47	1 (Reference)	1 (Reference)	1 (Reference)	
Uterine exposed	101/1042	7.98	1.39(1.01,1.93)	1.35(0.98,1.87)	1.32(0.95,1.83)	
Childhood exposed	601/5766	8.69	1.00(0.65,1.55)	0.97(0.63,1.51)	0.96(0.62,1.49)	

Model 1^a^: Adjusted for age

Model 1^b^: Adjusted for age, and gender

Model 1^c^: Same as the Model 1^b^

Model 2^a^: Included covariates in model 1a and further adjusted for education (junior high school or below, senior high school or above), smoking (current, never/former), drinking (current, never/former), and physical activity (current, never/former)

Model 2^b^: Included covariates in model 1b and further adjusted for education (junior high school or below, senior high school or above), drinking (current, never/former), and physical activity (current, never/former)

Model 2^c^: Included covariates in model 1c and further adjusted for education (junior high school or below, senior high school or above), smoking (current, never/former), and physical activity (current, never/former)

Model 3^a^: Included covariates in model 2a and further adjusted for use of antihypertensive medications, use of hypoglycemic medications, and use of hypolipidemic medications

Model 3^b^: Included covariates in model 2b and further adjusted for use of antihypertensive medications, use of hypoglycemic medications, and use of hypolipidemic medications

Model 3^c^: Included covariates in model 2c and further adjusted for use of antihypertensive medications, use of hypoglycemic medications, and use of hypolipidemic medications

## Discussion

Based on the prospective cohort, we found that exposure to Chinese famine during fetal life was associated with a higher risk of CVD in patients with MS. The results might help to elucidate the pathogenesis of CVD in MS individuals and emphasize the importance of adequate nutrition during the fetal period.

### Compared with other studies

To our knowledge, the effect of famine exposure on CVD risk in MS patients has not been evaluated, but studies confirmed that fetal exposure to famine increases CVD risk in those with the component of MS. A cross-sectional study found that the association between early life famine exposure and adult CVD risk appears to be stronger in overweight than in normal individuals [26]. Other studies showed that fetal exposure to famine exacerbates the adverse effect of hypertension on CVD, especially in individuals with central obesity [6, 27]. Zhang et al. found that exposure to famine, especially during fetal life, exacerbates the association between hyperglycemia and CVD. All these results support our research results to a certain extent.

### Mechanism

Although the mechanisms underlying this association between uterine famine exposure and adult CVD in MS individuals have not been elucidated, several mechanisms may explain the relationship. First, malnutrition early in life might affect structural changes in the cardiovascular system, and famine exposure during uterine life might lead to epigenetic changes, even with lifelong effects [28]. Second, findings from the Dutch Famine Study suggest that prenatal exposure to famine increases the preference for high-fat foods and a high prevalence of dyslipidemia [29], which in turn increases the risk of CVD [30]. Third, several studieshave shown that confirmed the interaction between hypertension, hyperglycemia and famine on increase the risk of CVD [6, 7]. And hypertension and diabetes are components of MS, the risk of CVD might be significantly increased by experiencing fetal famine exposure in the MS individuals.

### Stratification analysis

After stratification for sex, the results remained statistically significant in female, but not in male. Previous studies proved that female hormonal complex and CVD risk was deeply intertwined. For example, sex hormones have vasodilating properties that protective effect of blood vessel wall and estrogen appears to prevent coronary artery spasms. The major CVD risk factors were changed by loss of estrogen (the lipid profile changes with menopause, becoming more atherogenic with increase of low protein cholesterol levels) [31].In addition, traditional Chinese values that favor boys and discriminate against girls may also be partly to blame. Most studies were conducted in times of food shortages when families tend to allocate food and other resources to their sons than to their daughters, helping more male infants who suffer from famine to survive and grow after birth, so the female population is more affected by famine and at greater risk of CVD in adulthood [32].

After stratification for smoking, the results remained statistically significant in smoker, but not in non-smoker. One possible explanation for these relationships is that smoking might mediate CVD risk through shared pathophysiology, including dyslipidemia, hyperlipidemia, and abdominal obesity. Prevention strategies to reduce the burden of CVD therefore require the maintenance of a healthy lifestyle.

### Advantages and limitations

The main advantages of our study are its prospective nature, the long follow-up time, and the large sample size. In addition, CVD event information was collected through the health insurance system rather than self-reporting, so the data was more reliable and realistic. However, the study has some limitations. All participants in Kailuan cohort were employees from Kailuan Group, an industry dominated by coal mines with mostly male employees (75.53%), so it might be difficult in extrapolating to females or general individuals. In addition, due to the lack of exact famine exposure information in the current study, the grouping was based on year of birth, which might have classified those who did not suffer from famine into the uterine famine exposed group, resulting in a weaker famine effect. A proportion of participants in the uterine famine exposure group had also been exposed to famine in early childhood, which might have a synergistic effect on CVD. The lack of data in this study related to poor nutrition in the maternal diet, exposure to harmful agents, or risky lifestyle factors, might have confounded the findings, all of which should be explored in future studies. Thus, our findings are only suggestive of this association and need to be supported by further investigations data from famine individuals in other countries.

### Conclusion

In summary, we found that exposure to famine during fetal life in patients with MS is associated with a high risk of CVD in life.

## Data Availability

The data that support the findings of this study are available from [third party name] but restrictions apply to the availability of these data, which were used under license for the current study, and so are not publicly available. Data are however available from the corresponding author upon reasonable request and with permission of the corresponding author.

## Abbreviations

BMI: Body mass index
CI: Confidence interval
CVD: Cardiovascular disease
DBP: Diastolic blood pressure
FBG: Fasting blood glucose
HDL: High density cholesterol
HR: Hazard ratio
IDF: International diabetes federation
MI: Myocardial infarction
MS: Metabolic syndrome
SBP: Systolic blood pressure
WC: Waist circumference

## References

[1] Marcotte-Chenard A, Deshayes TA, Ghachem A, Brochu M (2019). Prevalence of the metabolic syndrome between 1999 and 2014 in the United States adult population and the impact of the 2007–2008 recession: an NHANES study. Appl Physiol Nutr Metab.

[2] He Y, Li Y, Bai G (2019). Prevalence of metabolic syndrome and individual metabolic abnormalities in China, 2002–2012. Asia Pac J Clin Nutr.

[3] Klongthalay S, Suriyaprom K (2020). Increased uric acid and life style factors associated with metabolic syndrome in thais. Ethiop J Health Sci.

[4] Mottillo S, Filion KB, Genest J (2010). The metabolic syndrome and cardiovascular risk a systematic review and meta-analysis. J Am Coll Cardiol.

[5] Ballantyne CM, Hoogeveen RC, McNeill AM (2008). Metabolic syndrome risk for cardiovascular disease and diabetes in the ARIC study. Int J Obes.

[6] Shi Z, Nicholls SJ, Taylor AW, Magliano DJ, Appleton S, Zimmet P (2018). Early life exposure to Chinese famine modifies the association between hypertension and cardiovascular disease. J Hypertens.

[7] Zhang Y, Ying Y, Zhou L, Fu J, Shen Y, Ke C (2019). Exposure to Chinese famine in early life modifies the association between hyperglycaemia and cardiovascular disease. Nutr Metab Cardiovasc Dis.

[8] Ekblom-Bak E, Halldin M, Vikstrom M (2021). Physical activity attenuates cardiovascular risk and mortality in men and women with and without the metabolic syndrome—a 20-year follow-up of a population-based cohort of 60-year-olds. Eur J Prev Cardiol.

[9] Zhang L, Guo Z, Wu M, Hu X, Xu Y, Zhou Z (2013). Interaction of smoking and metabolic syndrome on cardiovascular risk in a Chinese cohort. Int J Cardiol.

[10] Gowland RL (2015). Entangled lives: implications of the developmental origins of health and disease hypothesis for bioarchaeology and the life course. Am J Phys Anthropol.

[11] Burlina S, Dalfra MG, Lapolla A (2019). Short- and long-term consequences for offspring exposed to maternal diabetes: a review. J Matern Fetal Neonatal Med.

[12] Qin LL, Luo BA, Gao F, Feng XL, Liu JH (2020). Effect of exposure to famine during early life on risk of metabolic syndrome in adulthood: a meta-analysis. J Diabetes Res.

[13] Li Y, Li Y, Gurol ME (2020). In utero exposure to the Great Chinese Famine and risk of intracerebral hemorrhage in midlife. Neurology.

[14] Wang B, Cheng J, Wan H (2021). Early-life exposure to the Chinese famine, genetic susceptibility and the risk of type 2 diabetes in adulthood. Diabetologia.

[15] Xin X, Yao J, Yang F, Zhang D (2018). Famine exposure during early life and risk of hypertension in adulthood: a meta-analysis. Crit Rev Food Sci Nutr.

[16] Tao B, Yang P, Wang C (2021). Fetal exposure to the Great Chinese Famine and risk of ischemic stroke in midlife. Eur J Neurol.

[17] Wu Z, Jin C, Vaidya A (2016). Longitudinal patterns of blood pressure, incident cardiovascular events, and all-cause mortality in normotensive diabetic people. Hypertension.

[18] Wu S, Huang Z, Yang X (2012). Prevalence of ideal cardiovascular health and its relationship with the 4-year cardiovascular events in a northern Chinese industrial city. Circ Cardiovasc Qual Outcomes.

[19] Wang J, Perona JS, Schmidt-RioValle J, Chen Y, Jing J, Gonzalez-Jimenez E (2019). Metabolic syndrome and its associated early-life factors among Chinese and Spanish adolescents: a pilot study. Nutrients.

[20] Ding X, Li J, Wu Y (2021). Ideal cardiovascular health metrics modify the association between exposure to chinese famine in fetal and cardiovascular disease: a prospective cohort study. Front Cardiovasc Med.

[21] Jin C, Chen S, Vaidya A (2017). Longitudinal change in fasting blood glucose and myocardial infarction risk in a population without diabetes. Diabetes Care.

[22] Ma C, Pavlova M, Liu Y (2017). Probable REM sleep behavior disorder and risk of stroke: a prospective study. Neurology.

[23] Li W, Jin C, Vaidya A (2017). Blood pressure trajectories and the risk of intracerebral hemorrhage and cerebral infarction: a prospective study. Hypertension.

[24] Wu S, An S, Li W (2019). Association of trajectory of cardiovascular health score and incident cardiovascular disease. JAMA Netw Open.

[25] Schemper M, Wakounig S, Heinze G (2009). The estimation of average hazard ratios by weighted Cox regression. Stat Med.

[26] Li Y, Jaddoe VW, Qi L (2011). Exposure to the Chinese famine in early life and the risk of hypertension in adulthood. J Hypertens.

[27] Wang Y, Jin J, Peng Y, Chen Y (2021). Exposure to Chinese famine in the early life, adulthood obesity patterns, and the incidence of hypertension: a 22-year cohort study. Ann Nutr Metab.

[28] Heijmans BT, Tobi EW, Stein AD (2008). Persistent epigenetic differences associated with prenatal exposure to famine in humans. Proc Natl Acad Sci USA.

[29] Lussana F, Painter RC, Ocke MC, Buller HR, Bossuyt PM, Roseboom TJ (2008). Prenatal exposure to the Dutch famine is associated with a preference for fatty foods and a more atherogenic lipid profile. Am J Clin Nutr.

[30] Hidayat K, Du X, Shi BM, Qin LQ (2020). Foetal and childhood exposure to famine and the risks of cardiometabolic conditions in adulthood: a systematic review and meta-analysis of observational studies. Obes Rev.

[31] Santilli F, D'Ardes D, Guagnano MT, Davi G (2017). Metabolic syndrome: sex-related cardiovascular risk and therapeutic approach. Curr Med Chem.

[32] Lv S, Shen Z, Zhang H (2020). Association between exposure to the Chinese famine during early life and the risk of chronic kidney disease in adulthood. Environ Res.

